# Worse Self-Reported Hearing Ability Is Associated With Greater Perceived Physical and Mental Fatigability

**DOI:** 10.1093/geroni/igab046.595

**Published:** 2021-12-17

**Authors:** Theresa Gmelin, Kyle Moored, Frank Lin, Justin Golub, Mary Wojczynski, Robert Boudreau, Angeline Galvin, Nancy W Glynn

**Affiliations:** 1 University of Pittsburgh Graduate School of Public Health, Pittsburgh, Pennsylvania, United States; 2 University of Pittsburgh, Pittsburgh, Pennsylvania, United States; 3 Johns Hopkins University, Johns Hopkins University, Maryland, United States; 4 Columbia University, New York, New York, United States; 5 Washington University School of Medicine, Washington University School of Medicine, Missouri, United States; 6 University of Southern Denmark, Odense, Hovedstaden, Denmark

## Abstract

Older adults with hearing loss often report higher fatigue due to effortful listening. We evaluated whether self-reported hearing ability is associated with perceived physical and mental fatigability (a more sensitive measure than fatigue) using the Pittsburgh Fatigability Scale (PFS). Older adults (N=2,558) from the Long Life Family Study Visit 2 (71.5±11.4 years; 54.8% women) completed PFS and self-reported hearing ability (worse=[fair,poor,very poor,deaf] or better=[good, excellent]). Age-adjusted PFS Physical and Mental scores were 2.3 and 2.5 lower, respectively, for worse vs. better hearing (p<.0001). Generalized estimating equations adjusted for family-relatedness, site, age, sex, cognitive function (Mini-Mental State Examination), education, and self-reported health. Compared to individuals with better hearing, those with worse hearing had a 42% and 44% greater odds of physical (≥15) (CI:1.12-1.80,p=0.0042) and mental(≥13) (CI:1.13-1.84,p=0.0034) fatigability, respectively. These observed associations may potentially be explained via complex psychosocial and cognitive aging pathways (e.g. effortful listening) to be examined in future work.

